# Stretchable and Robust Candle-Soot Nanoparticle-Polydimethylsiloxane Composite Films for Laser-Ultrasound Transmitters

**DOI:** 10.3390/mi11070631

**Published:** 2020-06-28

**Authors:** Muhammad Faraz, Muhammad Awais Abbasi, Pilgyu Sang, Donghee Son, Hyoung Won Baac

**Affiliations:** Department of Electrical and Computer Engineering, Sungkyunkwan University, Suwon 16419, Korea; mfaraz@g.skku.edu (M.F.); awais@g.skku.edu (M.A.A.); pgsang@skku.edu (P.S.); daniel3600@skku.edu (D.S.)

**Keywords:** stretchable devices, functional nanomaterials, carbon nanoparticles, nanocomposite, photoacoustic

## Abstract

Considerable attention has been devoted to the development of nanomaterial-based photoacoustic transmitters for ultrasound therapy and diagnosis applications. Here, we fabricate and characterize candle-soot nanoparticles (CSNPs) and polydimethylsiloxane (PDMS) composite-based photoacoustic transmitters, based on a solution process, not just to achieve high-frequency and high-amplitude pressure outputs, but also to develop physically stretchable ultrasound transmitters. Owing to its non-porous and non-agglomerative characteristics, the composite exhibits unique photo-thermal and mechanical properties. The output pressure amplitudes from CSNPs–PDMS composites were 20–26 dB stronger than those of Cr film, used as a reference. The proposed transmitters also offered a center frequency of 2.44–13.34 MHz and 6-dB bandwidths of 5.80–13.62 MHz. Importantly, we characterize the mechanical robustness of CSNPs–PDMS quantitatively, by measuring laser-damage thresholds, to evaluate the upper limit of laser energy that can be ultimately used as an input, i.e., proportional to the maximum-available pressure output. The transmitters could endure an input laser fluence of 54.3–108.6 mJ·cm^−2^. This is 1.65–3.30 times higher than the Cr film, and is significantly higher than the values of other CSNPs–PDMS transmitters reported elsewhere (22–81 mJ·cm^−2^). Moreover, we characterized the strain-dependent photoacoustic output of a stretchable nanocomposite film, obtained by delaminating it from the glass substrate. The transmitter could be elongated elastically up to a longitudinal strain of 0.59. Under this condition, it maintained a center frequency of 6.72–9.44 MHz, and 6-dB bandwidth ranges from 12.05 to 14.02 MHz. We believe that the stretchable CSNPs–PDMS composites would be useful in developing patch-type ultrasound devices conformally adhered on skin for diagnostic and therapeutic applications.

## 1. Introduction

Photoacoustic transmitters for generating high-amplitude pulsed ultrasound have been developed in either planar or focal configuration, the latter of which allows laser-generated focused ultrasound (LGFU), which is widely used for such applications as histotripsy, drug delivery, sonothrombolysis, and lithotripsy. As effective alternatives to their conventional piezoelectric counterparts, photoacoustic transmitters have been used, taking advantage of their high pressure amplitude, in the order of MPa, and their high frequency spectrum together with broad bandwidth. Their frequency spectrum ranges over tens of MHz (even up to hundreds of MHz), which is determined by an input laser pulse profile (mostly with a duration ranging from a few to tens of ns, to allow high-energy laser pulse) and light-absorber distribution within a transmitter. The input laser pulses can be converted into short ultrasonic pulses, as the output from the photoacoustic transmitters (e.g., nano-composite), comprising light-induced heating elements (conducting fillers) and a thermal expansion medium (e.g., elastomeric polymer) [1,2,3,4,5,6,7]. 

As transmitter materials, a variety of nanostructures, including graphite, metallic films [8], gold nanoparticles and nanostructures [2,9,10], carbon black (CB) [7,11,12], carbon nanotubes (CNT) [4,5,6], and carbon nanofibers [13], were exploited to enhance the performance of photoacoustic transmitters. Most of these materials were designed to enhance optical absorption and thermal expansion. Due to the low specific heat capacity of nanoparticles, these can efficiently and rapidly transfer thermal energy into the surrounding medium for thermal expansion. For thermal expansion, an elastomeric polymer with a large coefficient of thermal expansion (β) is also greatly desired in order to generate high-pressure acoustic output. Polydimethylsiloxane (PDMS) has been extensively utilized for this purpose (β = 0.92 × 10^−3^ K^−1^) [5]. The photoacoustic conversion efficiency of nanocomposite transmitters, comprising carbon-based nanoparticles and PDMS, is exceptional as compared to those of other materials [5,13,14], e.g., CNT-PDMS films exhibit two orders-of-magnitude higher efficiency than the Cr film. The relation between absorption, volume fraction and thickness of CNTs has been analyzed [15], using finite-difference time-domain (FDTD) simulations. 

Recently, stretchable devices have attracted great attention as favorable alternatives to conventional rigid devices, especially for next-generation healthcare and biomedical applications. These devices are capable of accommodating mechanical deformation during their operation, which makes them suitable for personalized healthcare systems, wearable displays and implantable devices [16,17,18,19,20,21,22,23,24,25,26]. A material-based approach, which includes material synthesis and fabrication, is considered to be a promising method for developing intrinsically stretchable devices [27]. Owing to their electrical and mechanical properties, nanocomposites consisting of conducting fillers and elastomers are preferred over traditional electronic materials [28,29,30,31]. Previously, CB, CNTs and reduced graphene oxides were utilized as conducting fillers for producing stretchable nanocomposites [32,33,34,35,36]. As a composite matrix, the PDMS elastomer can be employed, as it can endure a significant level of mechanical strain, thus avoiding permanent deformation [27]. 

Among the carbon-based nanostructures, candle-soot nanoparticles (CSNPs), which are abundant carbonaceous substances, have gained significant attention due to the advantages of simple production, stability and cost effectiveness [37,38,39,40], although they have relatively poor robustness, confirmed with regard to their laser-induced damage threshold, and thus they have a limited output pressure strength. They have been utilized to build super-hydrophobic surfaces, flexible infrared nanosensors, and volumetric receivers [41,42,43]. CSNPs have also been employed to develop thin-film photoacoustic transmitters. Chang et al. [14,44] initially proposed such photoacoustic transmitters by transplanting spin-coated, pre-cured PDMS onto CSNPs using two glass slides, with a CSNPs-deposited slide facing toward the other slide (with a PDMS coating). Later, they improved the fabrication process by using low-viscosity PDMS (i.e., toluene-added PDMS), which was directly coated on the CSNPs deposited on a glass slide [45,46]. Li et al. [47] constructed LGFU transmitters using an optical fiber and a photoacoustic lens coated in CSNPs. The lens was dipped in a PDMS pool at a very low speed to form a nanocomposite structure. Moreover, CSNPs have also been coated directly on the optical fiber, and on an epoxy-based micro-lens bonded to an optical fiber, to develop fiber-based miniature transmitters that incorporate CSNPs–PDMS composites [48,49]. 

Despite the advantages of CSNPs–PDMS composite transmitters, they have a few disadvantages that need to be addressed. Firstly, during CSNPs deposition, the substrate should endure a high-temperature candle flame (~800–1400 °C) for a few seconds, which may damage the substrate. Secondly, the adhesion between CSNPs and the glass substrate is usually very weak, as they do not have any molecular linking or bonding to a substrate, unlike CNTs developed using a chemical vapor deposition process. Due to this reason, most of the CSNPs are easily washed away from a substrate, or often form an agglomeration during a spin-coating or dip-coating process within the PDMS matrix, which leads to significant degradation of the transmitter’s performance. Importantly, previous CSNPs–PDMS composites have suffered from poor mechanical robustness against laser-induced damage, characterized by a damage threshold fluence of < 100 mJ·cm^−2^. This greatly limits their use to low-fluence applications only [14,44,45,46,47,48,49,50], not suitable for areas requiring high-pressure amplitudes, such as therapy. 

We demonstrate CSNPs (conducting fillers)–PDMS (elastomer) nanocomposite-based photoacoustic transmitters, which can generate strong pressure amplitudes and endure a high laser fluence input. We describe the fabrication process of the transmitters, followed by the characterization of their photoacoustic outputs. The results confirm that the CSNPs–PDMS composite transmitters fabricated on a planar glass substrate generates peak pressure amplitudes 20, 22, 23 and 26 dB higher than that of a reference Cr film. These transmitters offer center frequencies of 2.44–13.34 MHz, and 6-dB bandwidths of 5.80–13.62 MHz. Furthermore, we obtained a stretchable nanocomposite transmitter by delaminating a composite film from the glass substrate, and evaluated its performance. As the composite is made of elastomeric polymer (PDMS), the transmitter is readily stretchable. This stretchable nanocomposite exhibited a center frequency of 6.72–9.44 MHz and a 6-dB bandwidth range of 12.05–14.02 MHz, when stretched by 19 mm. Finally, we quantified the mechanical robustness by measuring the laser-induced damage threshold, which determines the maximum-absorption energy fluence that can be tolerated in producing the ultimate output pressure from our proposed transmitters with optical absorption of ~100%. As compared to the Cr reference film (measured damage threshold = 32.9 mJ·cm^−2^), the transmitters exhibited a 1.65- to 3.30-fold higher laser damage threshold (54.3−108.6 mJ·cm^−2^). Such values are significantly higher than the values previously reported using similar CSNPs–PDMS composites (e.g., 22.93 mJ·cm^−2^ [14] and 81 mJ·cm^−2^ [47]). 

## 2. Materials and Methods 

### 2.1. Fabrication Process

**Figure 1:**
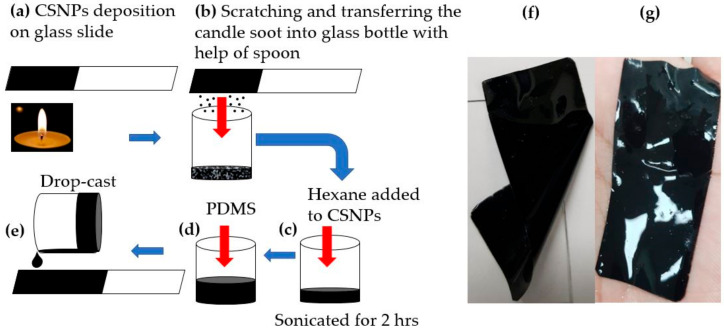
Fabrication process of candle-soot nanoparticles and polydimethylsiloxane (CSNPs–PDMS) nanocomposite. (**a**–**e**) Steps involved in the fabrication of the nanocomposite transmitter. (**f**,**g**) Examples of the stretchable nanocomposite films detached from a glass substrate.

A homogeneous dispersion method was utilized to prepare a solution of CSNPs uniformly blended in PDMS. A nanocomposite film was fabricated using a drop-casting process. The schematic diagram for fabrication is shown in Figure 1. A flame synthesis process [42] was employed to obtain CSNPs at room temperature using a paraffin wax candle with a diameter of 35 mm. A glass slide was continuously moved for several seconds within the flame core to allow uniform deposition of CSNPs. The deposited CSNPs were carefully harvested into a glass bottle by scratching the coated area with the help of a spoon. Then, we used hexane to mix with the CSNPs in a weight ratio of 20:1 (hexane:CSNPs). The mixture was sonicated for 2 h. The CSNPs–hexane solution was further mixed with PDMS prepolymer liquid and a curing agent in a ratio of 10:1 (Sylgard 184, Dow Corning, Midland, MI, USA). The ratio of PDMS to CSNPs was adjusted to 10:1, 20:1, 30:1 and 40:1 (named p10, p20, p30 and p40, respectively). Here, the hexane was used to reduce the viscosity of PDMS and thus ensure uniform mixture with the CSNPs. A planar transmitter was fabricated by drop-casting the CSNPs–hexane–PDMS solution onto a glass substrate, which was followed by a curing process at 90 °C for 30 min for evaporation of the hexane. Similarly, we also prepared the samples with different PDMS ratios with CSNPs (20:1, 30:1 and 40:1), using hexane with twice the amount of PDMS in each case. We denote each sample with a different ratio of PDMS to CSNPs as p10, p20, p30 and p40, respectively. Optical absorption for all films was almost 100% with no significant difference. Moreover, a stretchable nanocomposite film (denoted as p40d) was obtained by delamination from the glass substrate (Figure 1f,g).

**Figure 2:**
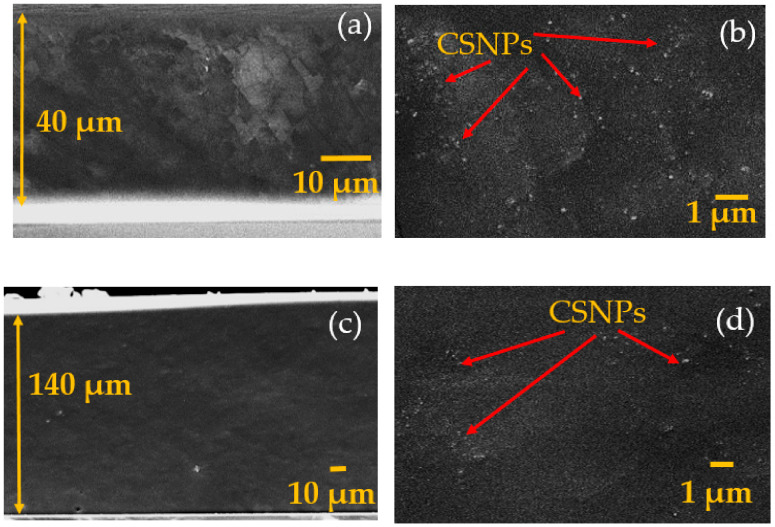
Scanning electron microscopy (SEM) photographs of the CSNPs–PDMS composite films (p10 and p40). Cross-sectional views for entire film thickness are shown in (**a**,**c**) for p10 and p40, respectively. Enlarged views for p10 and p40 are also shown in (**b**,**d**).

We confirmed the cross-sectional views of the fabricated CSNPs–PDMS composites by employing scanning electron microscopy (SEM) (JSM 7000F, JEOL, Tokyo, Japan). The CSNPs are spherical in shape (diameter around 40–50 nm) with a three-dimensional porous structure [14]. Figure 2 shows the images for p10 and p40. Figure 2a,c shows the entire thickness of the composite. Enlarged views are also shown in Figure 2b,d, which clearly confirm that p10 has relatively more CSNPs than p40. Figure 2c,d confirm the increased composite film thickness (140 μm), and the relatively sparse distribution of CSNPs within PDMS. For p20 and p30 (not shown here), CSNP densities were between p10 and p40. The composite film thicknesses for p10, p20, p30 and p40 were 40, 82, 126 and 140 μm, respectively. 

### 2.2. Measurement Setup for Output Pressure Waveforms

**Figure 3:**
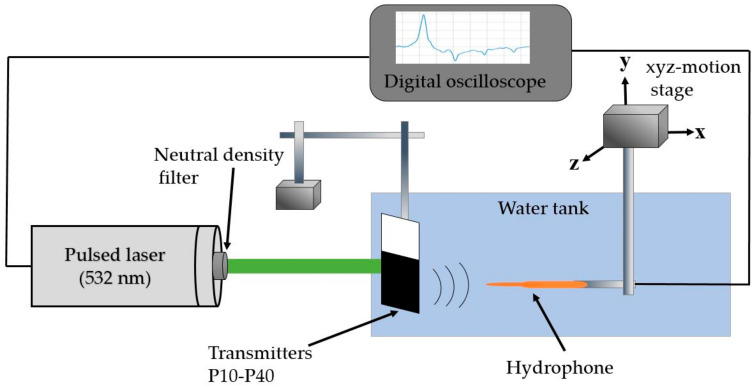
Experimental setup for characterization of the CNSPs–PDMS transmitters.

The proposed transmitters were characterized by utilizing the experimental setup depicted in Figure 3. The laser source was a *Q*-switched pulsed Nd:YAG laser (Litron Laser, Warwickshire, UK) (532-nm wavelength, 7-ns width, 10-Hz repetition rate and 8-mm beam diameter). The input laser energy to the transmitters was attenuated by utilizing neutral density filters. Laser irradiation was supplied to the transparent (planar) side of the transmitter. The photoacoustic waves generated by the transmitters were detected by using a polyvinylidene difluoride (PVDF) needle hydrophone with a 1-mm diameter (Precision Acoustics, Dorset, UK; 6-dB bandwidth of 20 MHz). The hydrophone was placed 2 mm away from the transmitters to satisfy a plane wave incidence configuration. The detected hydrophone signal was recorded by the digital oscilloscope (WaveSurfer 452, LeCroy, Chestnut Ridge, NY, USA) without any amplification. The waveforms generated by the transmitters were obtained by averaging 20 signal traces in a time-domain.

### 2.3. Measurement Setup for Laser-Induced Damage Threshold

The output pressure (*P*) from thin-film photoacoustic transmitters increases with incident optical fluence (*F*), Grüneisen coefficient (*Γ*) and optical absorption (*A*). It can be represented as the following:

(1) $$P=\frac{Г}{c}\;\frac{F}{\tau_{L}}\times \left(1-\eta \right)\times A,$$

where *c*, $\tau_{L}$ and η are the sound speed, the laser pulse duration, and the fraction of thermal energy sustained within heat absorbers after laser pulse duration, respectively [6]. Here, 1 − η means the fraction of thermal energy transferred to the surrounding PDMS. Although the output pressure can be enhanced with the input optical fluence and absorption by the nanocomposite, the laser-induced damage threshold (*F*_th_) determines an upper limit of such incident optical fluence. A higher optical input (*F* ≥ *F*_th_) causes physical damage to the transmitter. Hence, for a given optical absorption, the higher the damage threshold is, the stronger will be the maximum-available output pressure achieved by the transmitters.

**Figure 4:**
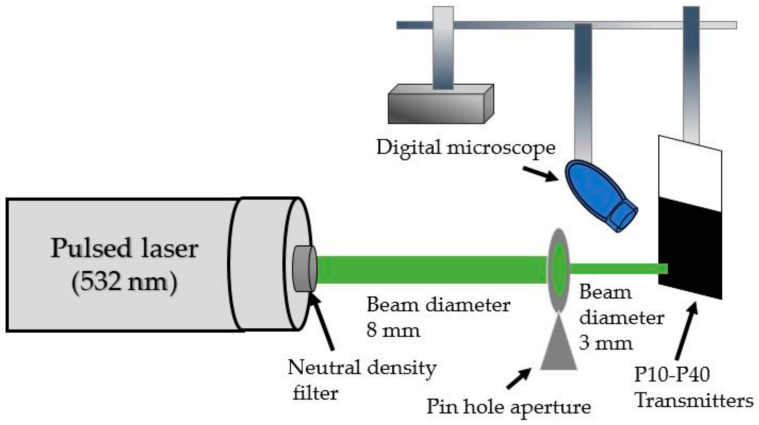
Experimental setup to measure the laser-induced damage threshold of CNSPs–PDMS transmitters.

The mechanical robustness of each transmitter was assessed by measuring the laser-induced damage threshold. Figure 4 shows the experimental setup. The same laser source already described in the previous setup was used to excite transmitters through a circular aperture with a 3-mm diameter. A digital microscope (Dino-Lite Pro, AnMo Electronics Corporation, Hsinchu, Taiwan) was fixed to capture optical images from the transparent side of the transmitters. We followed the measurement steps previously reported in [6], by increasing the incident laser fluence (mJ·cm^−2^) until a physical damage occurred.

## 3. Results and Discussions

### 3.1. Output Characteristics of CSNP–PDMS Composite Transmitters

**Figure 5:**
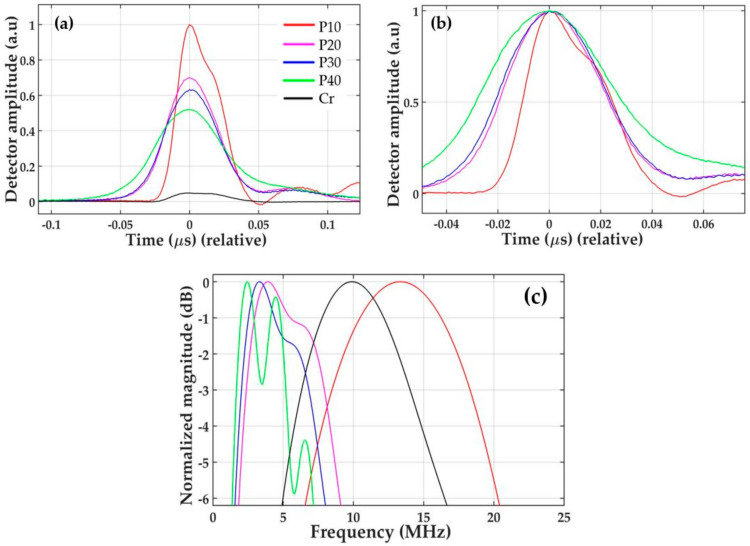
(**a**) Time-domain waveforms generated by the transmitters; (**b**) Comparison of normalized temporal pulse widths for the waveforms obtained in (**a**); (**c**) Frequency spectra obtained from the time-domain waveforms in (**a**).

We measured time-domain waveforms generated by the CSNPs–PDMS composites (p10, p20, p30 and p40) and the reference Cr film using the same laser energy of 1.5 mJ/pulse. The measured output pressure waveforms are shown in Figure 5a. The p10 composite generates an output pressure amplitude 20 times (26 dB) higher than that of the Cr film, and 3, 4 and 6 dB higher than those of the other three composites (p20–p40), respectively. The composites p20, p30 and p40 produce output pressures 23, 22 and 20 dB stronger than that of the Cr film, respectively. Such a strong output signal from the composites is mainly due to the high optical absorption and heating of the nanoscale absorbers, due to the low specific heat capacity (here, CSNPs), and the rapid transition of thermal energy to the surrounding PDMS with a high coefficient of thermal expansion. The higher performance from p10 as compared to other composites results from the higher concentration of CSNPs within the PDMS, as depicted in Figure 2b. It is clear that the CSNP particles in p40 are very sparsely distributed, as compared to p10. The overall film thickness also increases with the increase in the ratio of PDMS to CSNPs. The thinnest composite, p10 (40 µm), generates the strongest peak pressure, as compared to p20–p40, whereas the thickest composite, p40 (140 µm), generates the lowest peak amplitude as an output. For the transmitters p10–p40, we assume a uniform mixture (i.e., distribution) of CSNPs in PDMS, as they were prepared by the solution-based process with minimal agglomeration of CSNPs. This means that the optical absorption is uniform across the film’s thickness. Thus, p10 generates the highest peak pressure compared to the others, as the optical absorption in p10 can be tightly confined to a shorter depth. 

The measured waveforms resulted from the summation of forward-going (the transmitter to the detector) and backward-going (toward the glass substrate) waves, the latter of which is reflected by the glass substrate, changing its direction to forward. The depth of the optical absorption in the transmitter determines the delay in time between these propagating waves. The greater the absorption thickness is, the longer the time delay will be between them, which causes the output pulse width to broaden [51]. Normalized temporal positive pulse widths are depicted in Figure 5b. The widths were 36, 43, 45 and 58 ns for p10–p40, respectively, which is proportional to the round-trip delay path of each backward-going wave.

Figure 5c shows the frequency spectra obtained from the time-domain waveforms. These spectra include the bandwidth effect of the PVDF hydrophone. As the pulse broadening effect is caused by the increased amount of PDMS (increase in film thickness), the center frequency and the 6-dB bandwidth decreased from 13.34 to 2.44 MHz, and from 13.62 to 5.80 MHz, respectively. The characteristics for all transmitters are summarized in Table 1.

For the stretchable nanocomposite (p40d), we measured the time-domain waveforms for different stretching lengths. The original length of the nanocomposite without being stretched was found to be 32 mm, which was then stretched in 1-mm steps to the maximum of 19 mm before being damaged. The maximum-achievable longitudinal strain was 0.59 for the 19-mm elongation from the initial 32-mm length.

**Figure 6:**
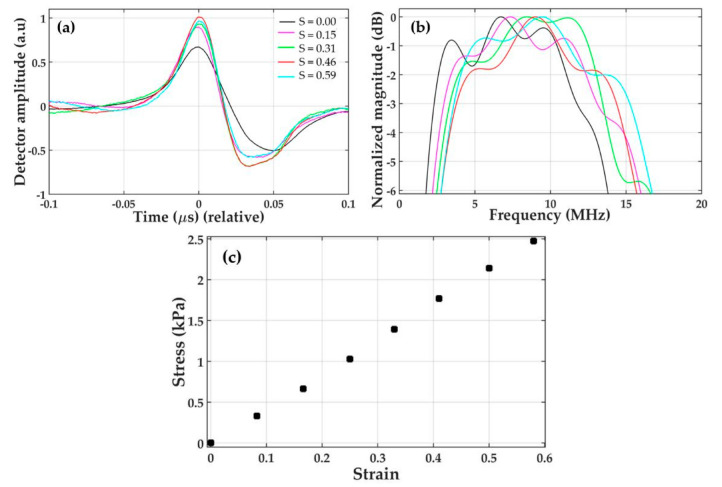
(**a**) Time-domain waveform generated by p40d transmitter under different strain values; (**b**) Frequency spectra measured from the time-domain waveforms in (**a**). (**c**) Stress-strain curve for p40d transmitter within its elastic limit.

The normalized output pressure waveforms for different strain (*S*) values (*S* is defined as the ratio of a longitudinal change in length to the original length) are represented in Figure 6a, where *S* = 0 corresponds to the case without any strain applied to the nanocomposite. After the elongation process, the nanocomposite film completely recovered its initial length. As the nanocomposite thickness was reduced by the stretching process, the pressure amplitude was enhanced. A 3.45-dB higher peak pressure amplitude was obtained at *S* = 0.46 (the condition before damage), as compared to that obtained at *S* = 0.15. However, the pressure at *S* = 0.15 was still 2.48-dB higher than that of the unstretched composite. The frequency spectra obtained from the time-domain waveforms are represented in Figure 6b. These shows that, with the increased strain from 0 to 0.59, the center frequency shifts towards the higher range, from 6.72 to 9.44 MHz.

Under strain, the CSNPs move in a direction perpendicular to the stretched length. This results in an increase in the volume fraction of CSNPs in the PDMS matrix. Thus, as the strain enhances, the CSNP particles being stretched come closer to each other, increasing the local concentration of CSNPs [52]. As shown in Figure 6a, this leads to an increased output amplitude and a decreased pulse width (FWHM), from 30 to 24 ns. A higher frequency response is also confirmed in Figure 6b, from 12.05 to 14.02 MHz. Therefore, the photoacoustic conversion efficiency can be increased with strain. This demonstrates that the strain-induced change in the absorption thickness, as well as an incremental change in the volume fraction of the nanoparticles, can be used as a way to tune the output frequency characteristics.

We obtained the stress-strain relation for the stretchable nanocomposite. In this case, the same sample (p40d) was utilized, with an original length of 12 mm and width of 25 mm. The composite was again stretched in 1-mm steps. Then, the corresponding force and stress were measured. For a longitudinal strain of 0.58 (i.e., the length extended from 12 to 19 mm as the maximum), the total applied force and stress were measured to be 0.74 N and 2.47 kPa, respectively. Figure 6c shows that stress is directly proportional to strain within the elastic limit of the nanocomposite. During the stretching process, the film also exhibited a necking effect, with the width reduced to 21.5 mm at a stress value of 2.14 kPa. The film underwent a brittle fracture with a clean cut when stretched beyond its elastic limit (*S* = 0.59) [53].

### 3.2. Mechanical Robustness for High-Energy Optical Absorption

**Figure 7:**
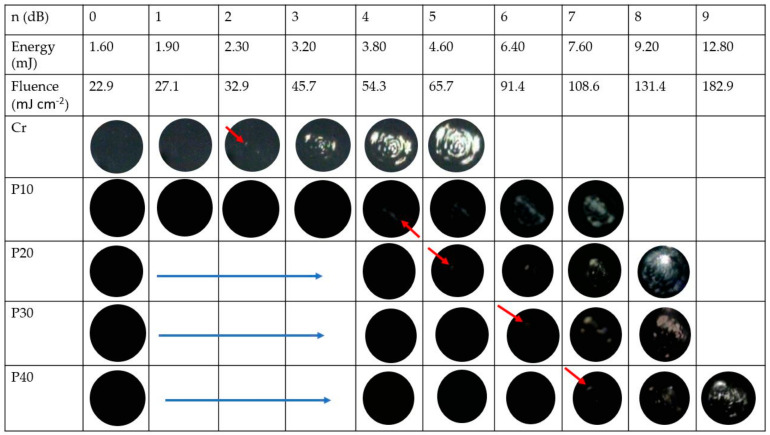
Photographs depicting the film surface of photoacoustic transmitters after pulsed laser irradiation. A given laser energy and fluence are shown in the first and second row, respectively. The red arrows indicate the starting point where ablated spots are firstly shown in each transmitter. No ablation was observed for p20–p40 with fluence less than 54.3 mJ·cm^−2^ (images not shown).

We measured the laser-induced damage threshold for our transmitters. Microscopic images for the transmitters are depicted in Figure 7 after pulsed laser irradiation. Here, an incident laser beam power represented by “*n* (dB)” was increased by a step of 1-dB. At *n* = 0, the obtained average laser beam power, pulse energy (*E*) and fluence (*F*) were 16.67 mW, 1.60 mJ and 22.9 mJ·cm^−2^, respectively. As shown in Figure 7, the laser-induced ablation for the reference Cr film started at *n* = 2 dB, corresponding to *E* = 2.30 mJ and *F* = 32.9 mJ·cm^−2^, whereas the ablation process for p10 started to appear when *E* was equal to 3.80 mJ (i.e., *F*_th_ = 54.3 mJ·cm^−2^). The laser damage threshold (*F*_th_) of p10 was 1.65-fold higher than that of the reference Cr film. For p20, the ablation started at *E* = 4.60 mJ, corresponding to *F*_th_ = 65.7 mJ·cm^−2^. The ablation threshold fluence for the p30 increased to 91.4 mJ·cm^−2^, which was 39% more than that of p20. Similarly, p40 presents a ~19 % higher damage threshold than p30, corresponding to *F*_th_ = 108.6 mJ·cm^−2^. For p10−p40 with ~100 % absorption, the maximum-absorption energy fluences (i.e., the product of optical absorption and the damage threshold) obtained were 54.3, 65.7, 91.4 and 108.6 mJ·cm^−2^, respectively. This confirms that the damage threshold increases with the ratio of the PDMS to CSNPs.

The p10 transmitter includes a higher concentration of CSNPs in PDMS than the others, leading to a shallower depth of optical absorption (i.e., a narrow temporal pulse width). Thus, under the same irradiation condition, the physical adhesion between the composite and the glass substrate for p10 would be exposed to a thermal load heavier than the other lower-density transmitters. For this reason, the p10 transmitter has the lowest damage threshold among transmitters compared here. Previously, the output pressure amplitude of p10 was two-fold stronger than that of p40 (Figure 5a). This means that the maximum-available pressure amplitude for p10 would be similar to that of p40, although p40 has a damage threshold double that of p10. However, we note that p10 has higher frequency characteristics than p40, despite the similar pressure amplitudes ultimately available for p10 and p40 at the upper limit of input laser energy. The laser damage thresholds of our composite transmitters range from 54.3 to 108.6 mJ·cm^−2^. This is similar to other values reported elsewhere for CSNPs–PDMS composite films (e.g., 81 mJ·cm^−2^ [47]) and CNT-PDMS composites fabricated by an electro-spinning process (120 mJ·cm^−2^) [54]. However, it is much lower than the 477 mJ·cm^−2^ obtained from the CNT–PDMS composite films containing CNTs grown via a chemical vapor deposition process [6].

Although the CSNPs–PDMS composite has an advantage in terms of its simple and low-cost fabrication process, its damage threshold still limits its possible application to diagnosis, not therapy. Due to the limited output pressure amplitude, previous CSNPs–PDMS composites were not used for in vivo applications requiring a significant tissue depth, of at least >1~2 cm. For cavitation-based therapy, photoacoustic lenses with low damage threshold values were often operated in a dual excitation manner with an additional transducer, or with externally injected seed bubbles [12]. In order to overcome such complexities in treatment, and to be useful for in vivo applications, the damage threshold of CSNPs–PDMS should be further increased. The output pressure amplitudes from these composite transmitters can also be increased by enhancing the photoacoustic energy conversion efficiency. In terms of composite formation, the spatial dispersion of carbon nanoparticles is essential in achieving favorable mechanical or electronic characteristics, and can thus improve such conversion efficiency [55,56,57].

## 4. Conclusions

We characterized the performances of the CSNPs–PDMS-based photoacoustic transmitters. We prepared the solution of CSNPs–PDMS, and analyzed the output pressure waveforms. The CSNPs–PDMS transmitters exhibited a center frequency of 2.44–13.34 MHz, and 6-dB bandwidth ranges from 5.80 to 13.62 MHz. As the ratio of PDMS to CSNPs increases, the amplitude of the acoustic signal decreases (also, the frequency response shifts towards a lower range). Importantly, we quantified the mechanical robustness with different ratios of PDMS to CSNPs, and then compared them with the reference Cr film. The CSNPs–PDMS transmitters not only produced significantly high-amplitude pressure outputs, but also endured a laser fluence up to 108.6 mJ cm^-2^, which is 36% higher than the previous CSNPs–PDMS transmitters [47]. Moreover, a stretchable nanocomposite transmitter was fabricated by delaminating the composite film from the glass substrate, and its performance was also analyzed. The stretchable nanocomposite provided a center frequency of 6.72–9.44 MHz, and 6-dB bandwidth ranges from 12.05 to 14.02 MHz, when a high longitudinal strain of 0.59 was applied with stretching from the initial length of 32 mm to 51 mm. The composite completely recovered after elongation, which suggests reversible use together with frequency tunability. We expect that the stretchable composites can be further developed for application in patch-type devices, that conformally adhere on skin for diagnostic and therapeutic applications.

## Figures and Tables

**Table 1:** Summarized characteristics of the CSNPs–PDMS and reference Cr film transmitters.

Transmitters	Normalized Peak Pressure (a.u)	Central Frequency (*F*_c_) (MHz)	*F*_-6dB_ Bandwidth	Pulse Width (ns)
p10	1.000	13.34	13.62	36
p20	0.698	3.90	7.17	43
p30	0.630	3.30	6.40	45
p40	0.518	2.44	5.80	58
Cr	0.048	9.80	11.50	43

## References

[1] Escoffre J.M., Bouakaz A. (2015). Therapeutic Ultrasound.

[2] Hou Y., Kim J.S., Ashkenazi S., O’Donnell M., Guo L.J. (2006). Optical generation of high frequency ultrasound using two-dimensional gold nanostructure. Appl Phys. Lett..

[3] Zhang S., Li F., Jiang X., Kim J., Luo J., Geng X. (2015). Advantages and challenges of relaxor-PbTiO3 ferroelectric crystals for electroacoustic transmitters–A review. Prog. Mater. Sci..

[4] Baac H.W., Ok J.G., Park H.J., Ling T., Chen S.L., Hart A.J., Guo L.J. (2010). Carbon nanotube composite optoacoustic transmitters for strong and high frequency ultrasound generation. Appl. Phys. Lett..

[5] Baac H.W., Ok J.G., Maxwell A., Lee K.T., Chen Y.C., Hart A.J., Zhen X., Euisik Y., Guo L.J. (2012). Carbon-nanotube optoacoustic lens for focused ultrasound generation and high-precision targeted therapy. Sci. Rep..

[6] Baac H.W., Ok J.G., Lee T., Guo L.J. (2015). Nano-structural characteristics of carbon nanotube-polymer composite films for high-amplitude optoacoustic generation. Nanoscale.

[7] Hou Y., Ashkenazi S., Huang S.W., O’Donnell M. (2008). An integrated optoacoustic transmitter combining etalon and black PDMS structures. IEEE Trans. Ultrason. Ferroelectr. Freq. Control.

[8] Biagi E., Margheri F., Menichelli D. (2001). Efficient laser-ultrasound generation by using heavily absorbing films as targets. IEEE Trans. Ultrason. Ferroelectr. Freq. Control.

[9] Tian Y., Wu N., Zou X., Felemban H., Cao C., Wang X. (2013). Fiber-optic ultrasound generator using periodic gold nanopores fabricated by a focused ion beam. Opt. Eng..

[10] Tian Y., Wu N., Sun K., Zou X., Wang X. (2013). Numerical simulation of fiber-optic photoacoustic generator using nanocomposite material. J. Comput. Acoust..

[11] Buma T., Spisar M., O’Donnell M. (2001). High-frequency ultrasound array element using thermoelastic expansion in an elastomeric film. Appl. Phys. Lett..

[12] Kim J., Chang W.Y., Lindsey B.D., Dayton P.A., Dai X., Stavas J.M., Jiang X. Laser-Generated-Focused Ultrasound Transmitters for Microbubble-Mediated, Dual-Excitation Sonothrombolysis. Proceedings of the IEEE International Ultrasonics Symposium (IUS).

[13] Hsieh B.Y., Kim J., Zhu J., Li S., Zhang X., Jiang X. (2015). A laser ultrasound transmitter using carbon nanofibers–polydimethylsiloxane composite thin film. Appl. Phys. Lett..

[14] Chang W.Y., Huang W., Kim J., Li S., Jiang X. (2015). Candle soot nanoparticles-polydimethylsiloxane composites for laser ultrasound transmitters. Appl. Phys. Lett..

[15] Bao H., Ruan X., Fisher T.S. (2010). Optical properties of ordered vertical arrays of multi-walled carbon nanotubes from fdtd simulations. Opt. Express.

[16] Lee W., Kobayashi S., Nagase M., Jimbo Y., Saito I., Inoue Y., Tanaka M., Yambe T., Sekino M., Yokota T. (2018). Nonthrombogenic, stretchable, active multielectrode array for electroanatomical mapping. Sci. Adv..

[17] Park S., Heo S.W., Lee W., Inoue D., Jiang Z., Yu K., Fukuda K., Jinno H., Sekino M., Yokota T. (2018). Self-powered ultra-flexible electronics via nano-grating-patterned organic photovoltaics. Nature.

[18] Choi M.K., Yang J., Kim D.C., Dai Z., Kim J., Seung H., Hyeon T., Kale V.S., Sung S.J., Lu N. (2018). Extremely vivid, highly transparent, and ultrathin quantum dot light-emitting diodes. Adv. Mater..

[19] Kim J., Shim H.J., Yang J., Choi M.K., Kim D.C., Kim J., Kim D.H., Hyeon T. (2017). Ultrathin quantum dot display integrated with wearable electronics. Adv. Mater..

[20] Yokota T., Zalar P., Kaltenbrunner M., Jinno H., Matsuhisa N., Kitanosako H., Someya T., Tachibana Y., Yukita W., Koizumi M. (2016). Ultraflexible organic photonic skin. Sci. Adv..

[21] Lee H., Choi T.K., Lee Y.B., Cho H.R., Ghaffari R., Wang L., Choi S.H., Lu N., Chung T.K., Hyeon T. (2016). A graphene-based electrochemical device with thermoresponsive microneedles for diabetes monitoring and therapy. Nat. Nanotechnol..

[22] Xu L., Gutbrod S.R., Bonifas A.P., Su Y., Sulkin M.S., Lu N., Lu C., Ying M., Webb C., Ameen A. (2014). 3D multifunctional integumentary membranes for spatiotemporal cardiac measurements and stimulation across the entire epicardium. Nat. Commun..

[23] Son D., Lee J., Qiao S., Ghaffari R., Kim J., Lee J.E., Yang S., Park M., Shin J., Kang K. (2014). Multifunctional wearable devices for diagnosis and therapy of movement disorders. Nat. Nanotechnol..

[24] Xu S., Zhang Y., Jia L., Mathewson K.E., Jang K.I., Kim J., Bhole S., Huang X., Chava P., Wang R. (2014). Soft microfluidic assemblies of sensors, circuits, and radios for the skin. Science.

[25] Kaltenbrunner M., Sekitani T., Reeder J., Yokota T., Kuribara K., Tokuhara T., Bauer S., Drack M., Graz I., Schwödiauer R. (2013). An ultra-lightweight design for imperceptible plastic electronics. Nature.

[26] Kim D.H., Lu N., Ma R., Kim Y.S., Kim R.H., Wang S., Islam I., Yu K.J., Kim T., Ying M. (2011). Epidermal electronics. Science.

[27] Kim D.C., Shim H.J., Lee W., Koo J.H., Kim D.H. (2019). Material-based approaches for the fabrication of stretchable electronics. Adv. Mater..

[28] Kim K.S., Zhao Y., Jang H., Lee S.Y., Kim J.M., Kim K.S., Ahn J.H., Kim P., Choi J.Y., Hong B.H. (2009). Large-scale pattern growth of graphene films for stretchable transparent electrodes. Nature.

[29] Bae S., Kim H., Lee Y., Xu X., Park J.S., Zheng Y., Balakrishnan J., Lei T., Kim H.R., Song Y.I. (2010). Roll-to-roll production of 30-inch graphene films for transparent electrodes. Nat. Nanotechnol..

[30] Moon G.D., Lim G.H., Song J.H., Shin M., Yu T., Lim B., Jeong U. (2013). Highly stretchable patterned gold electrodes made of au nanosheets. Adv. Mater..

[31] Choi C., Choi M.K., Liu S., Kim M.S., Park O.K., Im C., Kim J., Qin X., Lee G.J., Cho K.W. (2017). Human eye-inspired soft optoelectronic device using high-density mos 2-graphene curved image sensor array. Nat. Commun..

[32] Bhagavatheswaran E.S., Parsekar M., Das A., Le H.H., Wiessner S., Stöckelhuber K.W., Schmaucks G., Heinrich G. (2015). Construction of an interconnected nanostructured carbon black network: Development of highly stretchable and robust elastomeric conductors. J. Phys. Chem. C.

[33] Xu F., Wang X., Zhu Y., Zhu Y. (2012). Wavy ribbons of carbon nanotubes for stretchable conductors. Adv. Funct. Mater..

[34] Koo J.H., Song J.K., Kim D.H. (2019). Solution-processed thin films of semiconducting carbon nanotubes and their application to soft electronics. Nanotechnology.

[35] Liu Q., Chen J., Li Y., Shi G. (2016). High-performance strain sensors with fish-scale-like graphene-sensing layers for full-range detection of human motions. ACS Nano.

[36] Chen Z., Ren W., Gao L., Liu B., Pei S., Cheng H.M. (2011). Three-dimensional flexible and conductive interconnected graphene networks grown by chemical vapour deposition. Nat. Mater..

[37] Su Z., Zhou W., Zhang Y. (2011). New insight into the soot nanoparticles in a candle flame. Chem. Commun..

[38] Wei Z., Yan K., Chen H., Yi Y., Zhang T., Long X., Li J., Zhang L., Wang J., Yang S. (2014). Cost-efficient clamping solar cells using candle soot for hole extraction from ambipolar perovskites. Energy Environ. Sci..

[39] Zhang Z., Hao J., Yang W., Lu B., Tang J. (2015). Modifying candle soot with FeP nanoparticles into high-performance and cost-effective catalysts for the electrocatalytic hydrogen evolution reaction. Nanoscale.

[40] Zhang B., Wang D., Yu B., Zhou F., Liu W. (2014). Candle soot as a supercapacitor electrode material. RSC Adv..

[41] Deng X., Mammen L., Butt H.J., Vollmer D. (2012). Candle soot as a template for a transparent robust superamphiphobic coating. Science.

[42] Yuan L., Dai J., Fan X., Song T., Tao Y.T., Wang K., Xu Z., Zhang J., Bai X., Lu P. (2011). Self-cleaning flexible infrared nanosensor based on carbon nanoparticles. ACS Nano.

[43] Ni G., Miljkovic N., Ghasemi H., Huang X., Boriskina S.V., Lin C.T., Wang J., Xu Y., Rahman M.M., Zhang T. (2015). Volumetric solar heating of nanofluids for direct vapor generation. Nano Energy.

[44] Huang W., Chang W.Y., Kim J., Li S., Huang S., Jiang X. (2016). A novel laser ultrasound transmitter using candle soot carbon nanoparticles. IEEE Trans. Nanotechnol..

[45] Chang W.Y., Zhang X.A., Kim J., Huang W., Chang C.H., Jiang X. Photoacoustic Transduction Efficiency Evaluation of Candle Soot Nanoparticles/Pdms Composites. Proceedings of the IEEE 17th International Conference on Nanotechnology (IEEE-NANO).

[46] Chang W.Y., Zhang X.A., Kim J., Huang W., Bagal A., Chang C.H., Fang T., Wu H.F., Jiang X. (2018). Evaluation of photoacoustic transduction efficiency of candle soot nanocomposite transmitters. IEEE Trans. Nanotechnol..

[47] Li Y., Guo Z., Li G., Chen S.L. (2018). Miniature fiber-optic high-intensity focused ultrasound device using a candle soot nanoparticles-polydimethylsiloxane composites-coated photoacoustic lens. Opt. Express.

[48] Chang W.Y., Jiang X. A Fiber Optic Laser Ultrasound Transmitter using Candle Soot Nanoparticles/PDMS Composites. Proceedings of the IEEE 18th International Conference on Nanotechnology (IEEE-NANO).

[49] Kim J., Chang W.Y., Wu H., Jiang X. Optical Fiber Laser-Generated-Focused-Ultrasound Transmitters for Intravascular Therapies. Proceedings of the IEEE International Ultrasonics Symposium (IUS).

[50] Kim J., Kim H., Chang W.Y., Huang W., Jiang X., Dayton P.A. (2019). Candle-soot carbon nanoparticles in photoacoustics: Advantages and challenges for laser ultrasound transmitters. IEEE Nanotechnol. Mag..

[51] Joo M.G., Lee K.T., Sang P., Heo J., Park H.J., Baac H.W. (2019). Laser-generated focused ultrasound transmitters with frequency-tuned outputs over sub-10-MHz range. Appl. Phys. Lett..

[52] Nam T.H., Goto K., Nakayama H., Oshima K., Premalal V., Shimamura Y., Inoue Y., Naito K., Kobayashi S. (2014). Effects of stretching on mechanical properties of aligned multi-walled carbon nanotube/epoxy composites. Compos. Part A Appl. Sci. Manuf..

[53] McCabe J.F., Walls A.W. (2013). Properties used to characterize material. Applied Dental Materials.

[54] Poduval R.K., Noimark S., Colchester R.J., Macdonald T.J., Parkin I.P., Desjardins A.E., Papakonstantinou I. (2017). Optical fiber ultrasound transmitter with electrospun carbon nanotube-polymer composite. Appl. Phys. Lett..

[55] Gupta S., Sachan R., Bhaumik A., Narayan J. (2018). Enhanced mechanical properties of Q-carbon nanocomposites by nanosecond pulsed laser annealing. Nanotechnology.

[56] Liu J., Khan U., Coleman J., Fernandez B., Rodriguez P., Naher S., Brabazon D. (2016). Graphene oxide and graphene nanosheet reinforced aluminium matrix composites: Powder synthesis and prepared composite characteristics. Mater. Des..

[57] Yin J., Chang R., Shui Y., Zhao X. (2013). Preparation and enhanced electro-responsive characteristic of reduced graphene oxide/polypyrrole composite sheet suspensions. Soft Matter.

